# Artificial intelligence based predictive models for early sepsis detection in intensive care units: a scoping review

**DOI:** 10.3389/fdgth.2026.1794922

**Published:** 2026-05-21

**Authors:** Mariana González Garcés, Juanita Valencia García, Camilo Jiménez Triana, Jerónimo Cárdenas Montoya, Erwin Hernando Hernández Rincón

**Affiliations:** 1 Faculty of Medicine, Universidad de La Sabana, Chía, Colombia; 2 Department of Family Medicine and Public Health, Universidad de La Sabana, Chía, Colombia

**Keywords:** artificial intelligence, early detection, intensive care units, machine learning, predictive models, sepsis

## Abstract

**Background:**

Early detection of sepsis in intensive care units remains a major clinical challenge. Artificial intelligence based predictive models have emerged as promising tools to support early identification of sepsis, yet their clinical readiness and methodological robustness remain heterogeneous.

**Objective:**

To map and critically synthesize the available evidence on artificial intelligence based predictive models for early sepsis detection in intensive care units.

**Methods:**

A scoping review was conducted following the PRISMA ScR framework. Multiple databases were systematically searched to identify studies developing or validating artificial intelligence based models for early sepsis detection in adult intensive care settings. Data were extracted on study design, data sources, model type, prediction horizon, validation strategies, and reported performance.

**Results:**

Thirty seven studies were included. Most models were developed using retrospective electronic health record data and relied on machine learning techniques, with limited external validation. Reported performance varied widely, and few studies addressed clinical implementation, interpretability, or integration into real time workflows.

**Conclusions:**

Although artificial intelligence based models show potential for early sepsis detection, substantial gaps remain regarding external validation, clinical integration, and real world applicability. Future research should prioritize methodological transparency and implementation focused evaluation.

## Introduction

Sepsis is a life-threatening condition characterized by a dysregulated host response to infection that leads to acute organ dysfunction and, in severe cases, progresses to septic shock, defined by persistent hypotension, elevated serum lactate levels, and the need for vasopressors to maintain adequate mean arterial pressure (1). Despite sustained advances in critical care medicine, sepsis remains a major global health problem. In 2020, an estimated 48.9 million cases and 11 million sepsis-related deaths were reported worldwide, underscoring its persistent contribution to morbidity and mortality and its disproportionate impact on intensive care units (ICUs) (2). Within these settings, sepsis and septic shock account for a substantial proportion of admissions and are associated with high mortality, particularly among patients with advanced organ dysfunction (3, 4) (Tables 1–3).

**Table 1:** qSOFA score.

Variable	Criteria	Points
Respiratory rate	Equal to or greater than 22 breaths per minute	1
Systolic blood pressure	Equal to or less than 100 mmHg	1
Mental status	Altered level of consciousness (Glasgow Coma Scale less than 15)	1

Interpretation: A score of 2 or more points is associated with an increased risk of mortality in patients with suspected infection.

**Table 2:** SIRS criteria.

Parameter	Threshold	Meets criterion
Temperature	Greater than 38 °C or less than 36 °C	Yes/No
Heart rate	Greater than 90 beats per minute	Yes/No
Respiratory rate	Greater than 20 breaths per minute or PaCO_2_ less than 32 mmHg	Yes/No
White blood cell count	Greater than 12 000 cells per microliter, less than 4 000 cells per microliter or more than 10 percent bands	Yes/No

Interpretation: SIRS is defined as the presence of two or more criteria in the setting of suspected or confirmed infection.

**Table 3:** NEWS score.

Parameter	3 points	2 points	1 point	0 points
Respiratory rate	Less than 8	9 to 11	12 to 20	21 to 24
Oxygen saturation	Less than 91 percent	92 to 93 percent	94 to 95 percent	Greater than or equal to 96 percent
Temperature	Less than 35 °C	—	35.1 to 36 °C	36.1 to 38 °C
Systolic blood pressure	Less than 90 mmHg	91 to 100 mmHg	101 to 110 mmHg	Greater than 110 mmHg
Heart rate	Less than 40 beats per minute	41 to 50 beats per minute	51 to 90 beats per minute	91 to 110 beats per minute
Level of consciousness	New confusion or decreased alertness	—	—	Alert

Interpretation: Higher NEWS scores indicate increased risk of clinical deterioration.

Timely identification of sepsis continues to represent a fundamental clinical challenge. Early manifestations are frequently nonspecific, physiological deterioration may evolve rapidly, and the precise temporal onset of sepsis is often difficult to define in routine clinical practice. Widely used clinical scores such as qSOFA, SIRS, and NEWS provide structured approaches to risk stratification but exhibit important limitations in sensitivity, specificity, and consistency across clinical contexts (5–7). Importantly, these tools were not designed to predict early sepsis but rather to identify patients who already exhibit physiological derangements or organ dysfunction, which may limit their utility for proactive intervention. Reliance on intermittent manual assessment and subjective clinical interpretation may therefore contribute to delays in diagnosis and delayed initiation of appropriate treatment (5).

Over the past decade, artificial intelligence and machine learning approaches have been increasingly proposed as solutions to these limitations. By enabling continuous analysis of electronic health record data, laboratory results, and high frequency physiological signals, AI-based models aim to capture complex and nonlinear patterns that may precede clinical recognition of sepsis. Numerous studies have reported high discriminatory performance, often summarized using the area under the receiver operating characteristic curve, and have suggested the ability to identify sepsis hours before conventional diagnosis (5, 8, 9). However, the extent to which these models provide true prospective prediction rather than detection of established disease remains unclear. In many studies, key predictors such as fever, tachycardia, hypoxia, or laboratory abnormalities may emerge close to or after sepsis onset, raising concerns regarding information leakage and limited temporal validity.

In recent years, an increasing number of studies have explored the application of advanced machine learning and deep learning techniques for early sepsis detection, incorporating large-scale electronic health record data, high-frequency physiological signals, and multimodal inputs. Recent models have reported promising performance and have begun to explore real-time implementation and clinical integration, although important challenges remain regarding generalisability, external validation, and clinical utility (8, 10, 11).

Beyond these conceptual challenges, the rapid expansion of AI-based sepsis research has been accompanied by substantial methodological and contextual heterogeneity. Studies vary widely in algorithmic paradigms, data sources, outcome definitions, prediction horizons, and validation strategies. Most models are developed using retrospective data from single institutions or high income countries, frequently with limited external or prospective validation. In addition, dimensions critical to real world deployment, including interoperability with electronic health record systems, workflow integration, clinician trust, regulatory oversight, and ethical considerations related to bias and equity, are inconsistently addressed. These factors contribute to a persistent gap between promising algorithmic performance and limited adoption in routine clinical practice.

In this context, a critically informed synthesis of the available evidence is needed, not only to map existing approaches but also to examine their methodological robustness, clinical relevance, and limitations. The objective of this scoping review is to map, characterize, and synthesize the literature on AI-based models for early sepsis detection in adult intensive care units. This review examines model architectures, input variables, outcome operationalization, performance metrics, validation strategies, and reported clinical applications, while explicitly highlighting sources of bias, risks of misinterpretation, and barriers to implementation. By situating current evidence within the broader clinical, methodological, and informatics landscape, this review aims to identify key research gaps and inform future directions toward the safe, effective, and equitable use of artificial intelligence in critical care.

## Methods

### Study design and methodological rationale

This study was designed as a scoping review to systematically map and critically examine the literature on artificial intelligence based models for early sepsis detection in adult intensive care units. A scoping review methodology was deliberately selected because the field is characterized by marked heterogeneity in study designs, model architectures, outcome definitions, prediction horizons, and validation strategies, which currently precludes meaningful quantitative synthesis or meta analysis. In this context, a scoping review enables comprehensive evidence mapping while supporting structured critical interpretation of methodological patterns and gaps.

The conduct and reporting of this review followed the methodological guidance of the Joanna Briggs Institute and adhered to the PRISMA ScR extension (12). This framework was chosen to ensure transparency and reproducibility while allowing analytical flexibility appropriate for emerging and methodologically diverse research domains such as clinical artificial intelligence.

The review protocol was prospectively registered in the Open Science Framework prior to initiation of the literature search. Protocol registration was undertaken to enhance methodological transparency and reduce the risk of selective reporting. The full protocol is available at https://osf.io/gw7ut.

### Eligibility criteria

We included original research studies and systematic reviews that evaluated artificial intelligence or machine learning models for early detection or prediction of sepsis related outcomes in adult intensive care unit populations. Eligible studies were required to assess models whose primary or secondary objectives included prediction of sepsis onset, septic shock, mortality, length of stay, or decision support related to sepsis management.

To enable critical synthesis beyond descriptive mapping, included studies were required to report essential methodological information, including the artificial intelligence model type, input variables, outcome definitions, performance metrics, and at least one limitation or validation consideration. Studies that did not provide sufficient methodological detail to support critical interpretation were excluded.

Studies conducted exclusively outside the intensive care unit setting, including emergency departments, general wards, or prehospital environments, were excluded. This restriction was applied intentionally to reduce contextual heterogeneity and to focus on clinical environments characterized by continuous physiological monitoring and high baseline sepsis risk. Pediatric and neonatal studies were excluded due to fundamental differences in sepsis pathophysiology, care pathways, and data generation processes.

### Information sources and search strategy

A comprehensive literature search was conducted in PubMed, Scopus, and Web of Science. These databases were selected to ensure broad coverage of biomedical, clinical, and interdisciplinary research at the intersection of artificial intelligence, medical informatics, and critical care.

The search strategy combined free text terms related to sepsis, artificial intelligence, machine learning, early detection, and intensive care units. Searches were restricted to studies published between January 2015 and June 2025. This temporal restriction reflects the period during which contemporary machine learning and deep learning methods became widely applied to electronic health record data and high frequency physiological signals in critical care settings. Earlier studies were excluded due to limited relevance to current analytical paradigms and data infrastructures.

The full search strategies for each database are presented in Table 4. Retrieved records were exported to a reference management system for deduplication and subsequently imported into the Rayyan platform to support structured and blinded screening (13).

**Table 4:** Search strategy.

Database	Search strategy
PubMed	(“sepsis” OR “septic shock”) AND (“artificial intelligence” OR “machine learning” OR “deep learning” OR “neural networks” OR “predictive model”) AND (“intensive care unit” OR “ICU”) AND [2015:2025(dp)]
Scopus	TITLE-ABS-KEY (“sepsis” OR “septic shock”) AND TITLE-ABS-KEY (“artificial intelligence” OR “machine learning” OR “deep learning” OR “neural network” OR “predictive model”) AND TITLE-ABS-KEY (“intensive care unit” OR “ICU”) AND PUBYEAR > 2014 AND PUBYEAR < 2026
Web of Science	TS = (“sepsis” OR “septic shock”) AND TS = (“artificial intelligence” OR “machine learning” OR “deep learning” OR “neural network” OR “predictive model”) AND TS = (“intensive care unit” OR “ICU”) AND PY = 2015–2025

Grey literature and conference proceedings were not systematically searched. This decision was made to prioritize peer reviewed studies with sufficient methodological detail to allow critical appraisal of model development, validation strategies, and clinical relevance. The potential risk of publication bias associated with this approach is acknowledged and considered in the interpretation of findings.

### Study selection

Study selection was conducted in two sequential stages. Two reviewers independently screened titles and abstracts to identify potentially eligible studies. Full text articles were subsequently reviewed to confirm eligibility. Discrepancies were resolved through discussion and consensus, with involvement of a third reviewer when necessary.

### Data extraction and analytical domains

Data extraction was performed using a structured extraction matrix developed in accordance with Joanna Briggs Institute guidance. Extracted variables included study characteristics, clinical setting, population features, artificial intelligence model architecture, input variables, outcome operationalization, prediction horizons, performance metrics, validation strategies, and reported clinical applications or limitations.

To directly address concerns regarding robustness and bias, additional extraction domains included validation type, temporal alignment between predictors and outcomes, use of clinical scores as labels or predictors, and discussion of interpretability or implementation considerations.

### Data synthesis approach

Evidence synthesis was conducted using thematic analysis. A hybrid analytical framework was applied, combining deductive coding based on predefined scoping review domains with inductive coding to capture emergent methodological and clinical patterns. Coding was performed independently by two reviewers, and a third reviewer assessed thematic coherence and analytical consistency.

Consistent with scoping review methodology, no formal risk of bias assessment tool was applied. However, methodological features directly relevant to model validity and clinical applicability, including validation strategy, outcome definition, and potential sources of bias such as information leakage, were systematically extracted and synthesized to support critical interpretation of the evidence.

## Results

### Evidence base and study designs

**Figure 1:**
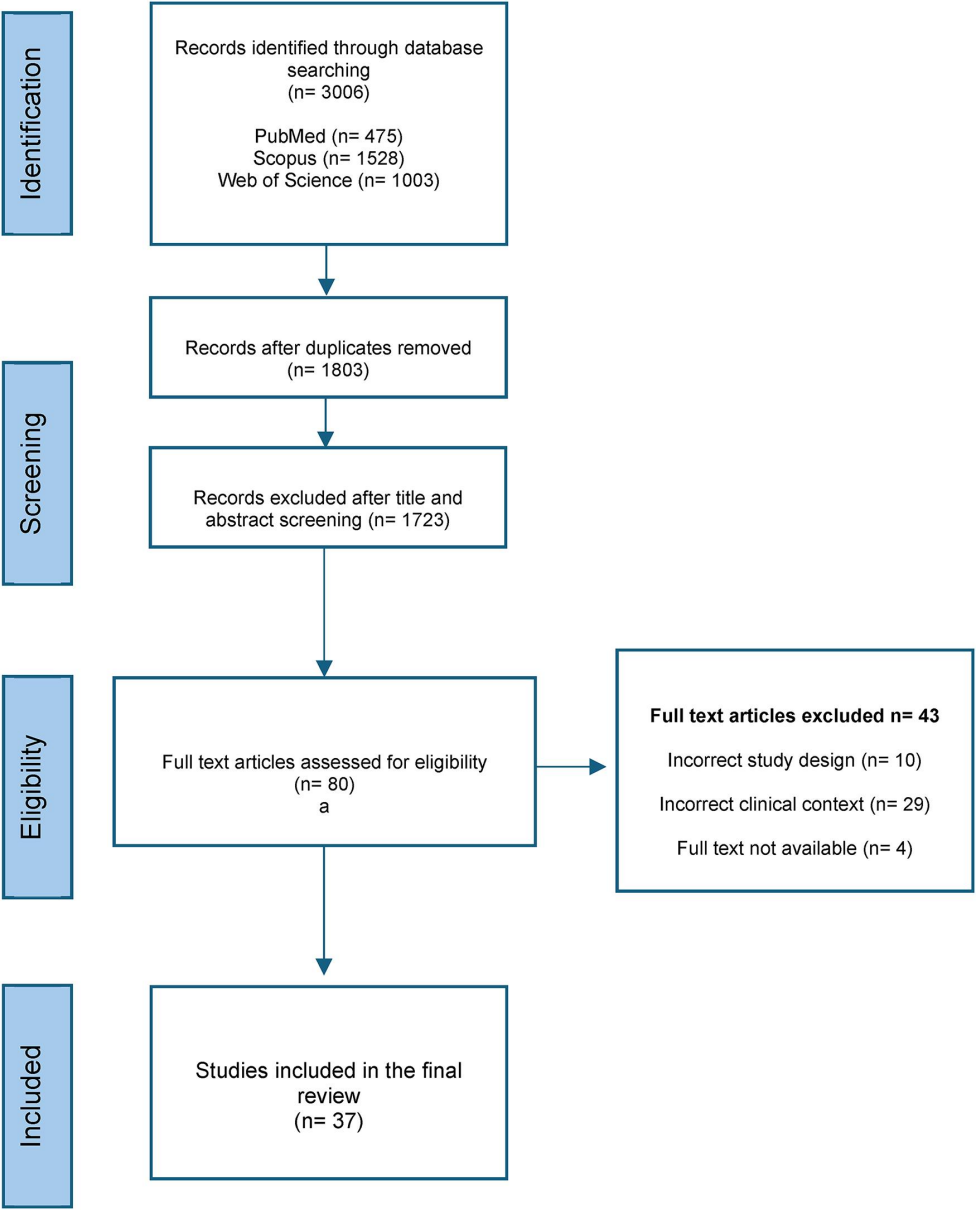
PRISMA 2020 flow diagram of the study selection process.

The literature search identified a total of 3,006 records across PubMed, Scopus, and Web of Science. After removal of duplicates, 1,803 unique records remained for screening. Following title and abstract review, 1,723 studies were excluded. Eighty full text articles were assessed for eligibility, of which 43 were excluded due to incorrect clinical context (*n* = 29), incorrect study design (*n* = 10), and full text not available (*n* = 4). A total of 37 studies were included in the final synthesis (14). The study selection process is presented in Figure 1, as detailed in the PRISMA flow diagram.

A structured summary of methodological and analytical characteristics across all included studies (*n* = 37) is presented in Table 5. This table summarizes key methodological and clinical features, including data sources, sample size, sepsis definitions, model types, prediction horizons, validation strategies, and reported performance. In addition, studies were classified according to their analytical intent as prospective prediction, near-onset detection, or operationally ambiguous.

**Table 5:** Summary of methodological and analytical characteristics across included studies (*n* = 37).

Domain	Summary across included studies	Representative references
Study design	The evidence base was predominantly composed of retrospective observational studies based on secondary analysis of clinical data. Prospective or semi-prospective evaluations were uncommon, and only a limited number of studies assessed models under real-time clinical conditions.	(15–22)
Primary data sources	Most studies used electronic health record data, including structured clinical variables, laboratory values, and intermittently recorded vital signs. A smaller subset incorporated continuous physiological monitoring, waveform signals, or multimodal inputs.	(16–18, 21–24)
Outcome operationalisation	Definitions of sepsis varied substantially across studies and included Sepsis-3 criteria, SIRS-based definitions, composite clinical rules, and administrative coding approaches. In several studies, clinical severity scores were also used as labels, inclusion criteria, or predictors.	(1, 6, 7, 15–17, 21, 25)
Analytical intent	Considerable conceptual ambiguity was identified between true prospective prediction and near-onset detection. Some models aimed to estimate sepsis risk before clinical recognition, whereas others appeared to identify already evolving physiological deterioration. In a subset of studies, the analytical intent remained operationally ambiguous because temporal alignment and outcome labelling were insufficiently specified.	(8, 15, 19, 26)
Model types	Traditional machine learning models were the most frequently reported, including Random Forest, XGBoost, logistic regression, support vector machines, decision trees, and ensemble approaches. Deep learning models, including LSTM, GRU-based architectures, convolutional neural networks, and temporal convolutional networks, were increasingly represented in more recent studies. Hybrid and multimodal approaches were also described.	(4, 16–18, 21–24, 27, 28)
Prediction horizon	Reported prediction windows ranged from approximately 3 to 24 h before labelled sepsis onset. Some studies used continuous or dynamically updated risk estimation rather than a fixed prediction horizon.	(16, 18, 19, 23, 28, 29)
Validation approach	Internal validation was the dominant strategy, usually based on random splits or temporally adjacent samples. External validation was infrequent, and temporal validation was inconsistently applied. Few studies explicitly assessed robustness to dataset shift or performance degradation over time.	(16, 17, 22, 30–33)
Performance reporting	AUROC was the most commonly reported metric, with many studies reporting apparently high discriminatory performance. However, clinically interpretable threshold-based metrics such as sensitivity, positive predictive value, false alert rates, and alert burden were inconsistently reported.	(4, 10, 16–18, 21, 22, 27)
Clinical implementation	Most studies proposed potential applications such as early warning systems, risk stratification, or decision support. Nevertheless, only a small subset evaluated implementation in real clinical workflows or examined prospective clinical impact. Overall, most models remained at proof-of-concept stage rather than clinically integrated deployment.	(5, 8, 10, 19, 20, 25)
Geographic distribution and generalisability	The included literature was concentrated in high-income and upper-middle-income settings, especially China and the United States. Representation from low- and middle-income contexts was limited, raising concerns regarding external validity, transferability, and equity.	(2, 3, 8, 31, 34)
Cross-cutting methodological concerns	Across the evidence base, high reported performance frequently coexisted with limited external validation, heterogeneous outcome definitions, risks of information leakage, limited interpretability, and sparse evaluation of real-world utility. This combination restricted confidence in clinical readiness.	(8, 10, 15, 31, 34)

This table provides a structured analytical summary of the 37 included studies and complements the model-category synthesis presented in Table 6. The table was designed to improve readability while preserving the main methodological and clinical patterns identified across the evidence base.

The included evidence base was dominated by retrospective observational studies using secondary analysis of electronic health record data (15–17, 21, 22). These studies typically relied on structured clinical variables, laboratory measurements, and intermittently recorded vital signs. A smaller subset incorporated high frequency physiological signals derived from continuous monitoring systems (18, 23, 24). Prospective or semi prospective study designs were uncommon, and only a limited number of studies evaluated models under real time clinical conditions (19, 20). As a result, the prevailing evidence reflects algorithmic performance in retrospective settings rather than demonstrated effectiveness in routine clinical care.

### Operational definitions of sepsis and temporal labeling

Considerable heterogeneity was observed in the operationalization of sepsis across included studies. Definitions of sepsis onset varied widely and included Sepsis 3 criteria, SIRS based definitions, combinations of clinical scores and laboratory thresholds, and administrative coding approaches (1, 15, 21, 25). In several studies, clinical severity scores such as SOFA, qSOFA, SIRS, or NEWS were used as outcome labels, inclusion criteria, or predictor variables (6, 7, 16, 17).

This variability introduced conceptual ambiguity regarding the temporal relationship between predictors and outcomes. In multiple studies, variables indicative of established organ dysfunction or physiological deterioration were temporally proximal to outcome labeling, raising concerns that reported model performance may reflect detection of ongoing sepsis rather than prospective prediction of future risk (8, 15, 26). Explicit differentiation between prediction and detection objectives was inconsistently reported, limiting interpretability of performance metrics and clinical relevance.

### Analytical intent classification: prediction vs. detection

A structured classification of the analytical intent of included studies revealed important heterogeneity in how early sepsis identification was conceptualised. Based on the temporal relationship between predictors and outcome definition, studies were categorised as prospective prediction, near-onset detection, or operationally ambiguous (Table 5).

A substantial proportion of studies classified as prospective prediction aimed to estimate the risk of sepsis several hours before clinical recognition, typically using prediction horizons ranging from 6 to 24 h. These models generally relied on longitudinal clinical data and were designed to support early intervention.

In contrast, studies classified as near-onset detection predominantly used predictors temporally close to the outcome, including physiological deterioration markers and laboratory abnormalities, suggesting that model outputs may reflect the identification of already evolving sepsis rather than true prospective prediction.

A smaller subset of studies was categorised as operationally ambiguous, as insufficient information was provided regarding temporal alignment, predictor selection, or outcome definition to clearly distinguish between predictive and detection objectives. This ambiguity limits interpretability and may contribute to overestimation of clinical utility in the existing literature.

### Artificial intelligence model types and validation strategies

A wide range of artificial intelligence model architectures were employed across studies. Traditional machine learning approaches such as Random Forest, XGBoost, logistic regression, support vector machines, decision trees, and ensemble methods were most frequently reported (16, 17, 21, 22, 27, 32). These models were predominantly applied to structured electronic health record data and were often selected for computational efficiency and relative interpretability (26, 30).

Deep learning architectures were increasingly represented in more recent publications and included recurrent neural networks such as LSTM and GRU based models, convolutional neural networks, and temporal convolutional networks designed to capture longitudinal or high dimensional physiological data (4, 18, 23, 28). Hybrid and multimodal models combining structured data with waveform signals or clinical notes were also described (18, 21, 24). However, across model types, explicit alignment between model complexity, data availability, and intended clinical use was often insufficiently articulated.

Validation strategies varied substantially. Most studies relied on internal validation using randomly split datasets or temporally adjacent samples (16, 17, 21, 22). External validation across independent institutions or health systems was infrequent (30–32). Temporal validation was inconsistently applied, and few studies explicitly addressed robustness to dataset shift or performance degradation over time (31, 33). Consequently, the strength of evidence supporting generalizability and real world reliability remained limited.

### Performance metrics and clinical interpretability

The area under the receiver operating characteristic curve was the most commonly reported performance metric. Machine learning models frequently reported AUROC values ranging from approximately 0.81 to 0.99 (16, 17, 21, 22, 27, 32), while deep learning models reported values up to 0.93 (4, 18). Prediction horizons ranged from three hours to twenty four hours prior to reported sepsis onset.

In contrast, clinically actionable performance measures such as sensitivity at predefined thresholds, positive predictive value, negative predictive value, and false alert rates were inconsistently reported (8, 10). In several studies, high AUROC values were presented without accompanying analysis of threshold selection or alert burden, limiting assessment of clinical utility in real world environments (8).

### Clinical implementation and real world impact

Most studies proposed potential clinical applications for their models, including early warning alerts, risk stratification, and decision support for clinicians (5, 8, 25). However, only a small subset of studies evaluated implementation within real clinical workflows or assessed prospective clinical impact (19, 20). Reports of improved outcomes such as reduced mortality or length of stay were limited and context dependent (20).

Overall, the maturity of deployment across the evidence base remained low, with most models positioned at the level of algorithmic proof of concept rather than clinically integrated systems (8, 10).

### Geographic concentration and implications for generalizability

**Figure 2:**
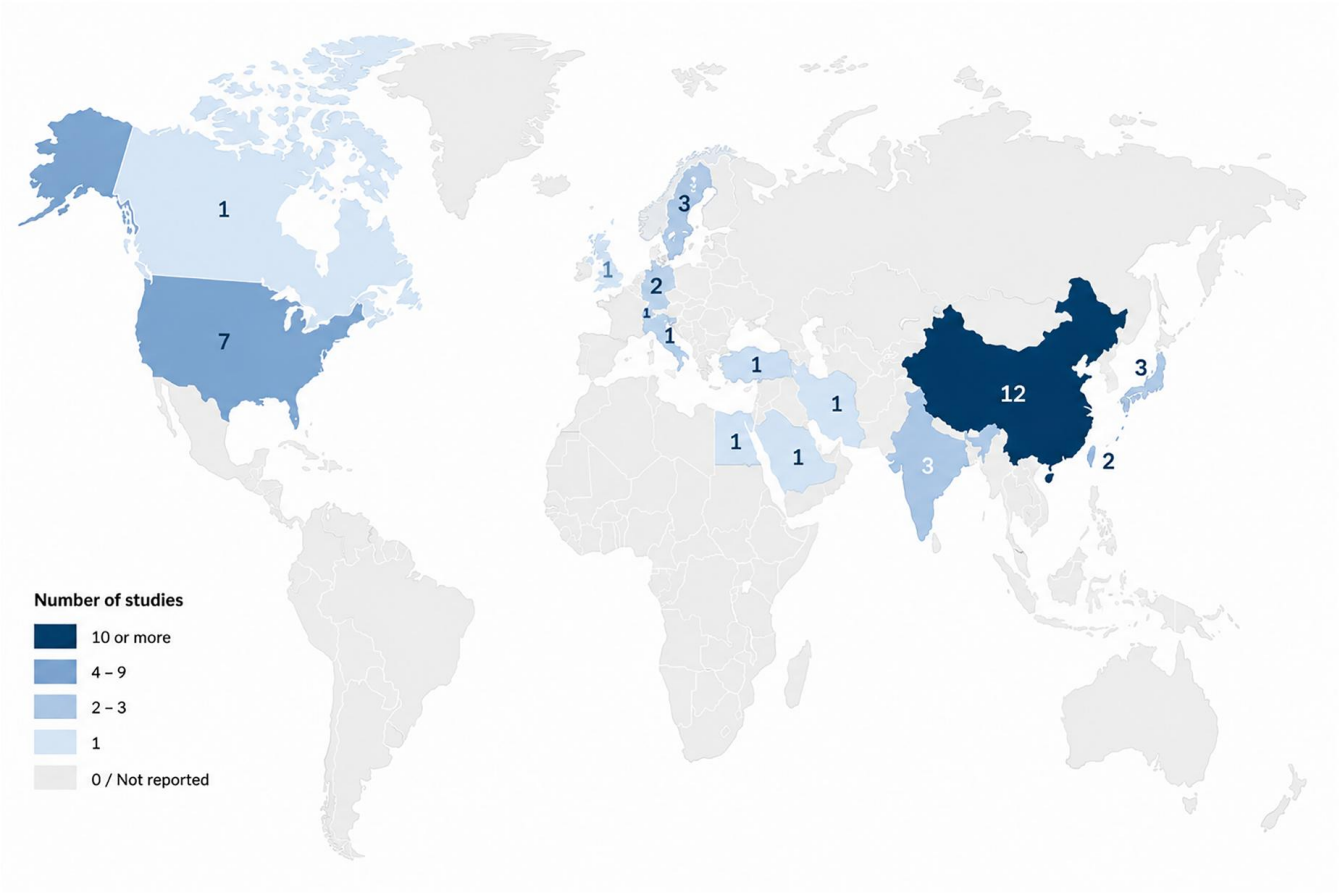
Geographic distribution of the studies included in this scoping review. The map illustrates the geographic distribution of the 37 studies included in this scoping review. China accounted for the largest number of studies, followed by the United States, with additional contributions from several countries in Europe, Asia, the Middle East, and North America. A smaller number of studies originated from Sweden, India, South Korea, Switzerland, and Taiwan, while single studies were identified from Italy, the United Kingdom, Iran, Saudi Arabia, the Netherlands, Egypt, Canada, Germany, Qatar, and Turkey. For studies conducted across multiple countries, each represented country was counted once to reflect geographic representation rather than the number of unique publications. Consequently, country-level counts may exceed the total number of included studies (*n* = 37). Map created using Natural Earth (https://www.naturalearthdata.com/about/terms-of-use/).

The geographic distribution of included studies was highly concentrated in high income and upper middle income countries, particularly China and the United States (2, 3) (Figure 2). Representation from low and middle income regions was limited. This concentration raises concerns regarding external validity and equity, as models developed in technologically mature intensive care environments may not generalize to settings with different data availability, infrastructure, or care pathways (2, 8).

### Integrated synthesis of methodological strengths and limitations

Across the included literature, several consistent patterns were identified. High reported discriminatory performance frequently coexisted with limited external validation, inconsistent outcome definitions, and limited assessment of clinical utility (8, 15, 31). Conceptual ambiguity regarding prediction vs. detection was commonly observed, alongside heterogeneous validation strategies and sparse evaluation in real world clinical settings.

Table 6 provides an integrated analytical synthesis of the included studies, comparing artificial intelligence model categories across key methodological and clinical dimensions. Rather than serving as a descriptive summary, this synthesis delineates differences in outcome operationalization, prediction vs. detection intent, validation approaches, reported performance metrics, and clinical actionability, highlighting recurring patterns related to generalizability, temporal alignment, and real world evaluation.

**Table 6:** Aggregated synthesis of artificial intelligence models for early sepsis detection in ICUs.

Aspect	Traditional machine learning models	Deep learning models	Hybrid or multimodal models	Proprietary clinical decision support systems
Model category	Random Forest, XGBoost, logistic regression, support vector machines, decision trees	LSTM, GRU D, convolutional neural networks, temporal convolutional networks, deep neural networks	Combination of machine learning and deep learning architectures using multiple data modalities	Vendor developed clinical decision support systems integrated into ICU platforms
Primary data sources	Structured electronic health record data, intermittently recorded vital signs, laboratory values, clinical severity scores	Longitudinal electronic health record data, high frequency physiological signals, waveform data	Structured EHR data combined with laboratory values, physiological waveforms, and clinical notes	Real time intensive care unit monitoring data integrated with clinical information systems
Outcome operationalization	Sepsis defined using Sepsis 3 criteria, SIRS based definitions, or composite clinical rules; severity scores often used as labels or predictors	Time dependent clinical criteria or score based labeling of sepsis onset	Composite outcome definitions integrating clinical criteria and laboratory thresholds	Institution specific operational definitions of sepsis and septic shock
Prediction vs. detection intent	Frequently unclear; several models likely identify established sepsis rather than predict future onset	Variable; some models aim at early risk estimation, others likely detect near onset physiological deterioration	More explicit intent to estimate risk, but temporal separation often insufficiently defined	Primarily detection oriented with early alert intent rather than long horizon prediction
Study design	Predominantly retrospective observational studies	Retrospective studies using large datasets	Model development studies and retrospective analytical studies	Prospective studies and pilot implementations
Validation strategy	Mostly internal validation; limited temporal validation; external validation rare	Internal validation common; some temporal validation; external validation uncommon	Internal validation with limited external validation	Prospective validation in selected studies; limited multicenter evaluation
Prediction horizon	Three to twenty four hours prior to labeled sepsis onset	Six to twenty four hours prior to labeled onset; some continuous risk estimation	Six to twelve hours or continuous risk updating	Six to thirteen hours before clinical recognition
Performance metrics reported	AUROC predominantly reported; inconsistent reporting of sensitivity, specificity, or predictive values	AUROC predominantly reported; limited use of clinically interpretable secondary metrics	AUROC with occasional reporting of sensitivity and specificity	AUROC with limited reporting of secondary metrics
Clinical actionability	Limited; alert thresholds and false alarm burden rarely evaluated	Uncertain; model complexity limits transparency and threshold selection	Potentially moderate; workflow integration rarely tested	Higher than other categories; some assessment of clinical impact
Key methodological concerns	High risk of information leakage; outcome definitions closely aligned with predictor variables; limited generalizability	Limited interpretability; dependence on advanced monitoring infrastructure; unclear clinical decision windows	Increased model complexity without proportional validation; residual information leakage risk	Limited algorithm transparency; vendor dependence; uncertain transferability
References	(9, 11, 16, 17, 21, 27, 35, 36)	(18, 22, 24, 28, 37)	(18, 24, 28, 37)	(19, 20, 26, 29, 30, 38)

## Discussion

This scoping review synthesizes the current evidence on artificial intelligence based approaches for early sepsis detection in intensive care units and demonstrates that, despite rapid growth in published studies, the field remains methodologically fragmented. While advances in computational techniques have expanded analytical capabilities, this expansion has not been matched by consistent gains in validation rigor, clinical interpretability, or real world implementation (8, 15, 39–49).

A central finding of this review is the persistent conceptual ambiguity between sepsis prediction and sepsis detection. As summarized in Table 6, many models described as early prediction systems rely on predictors that are temporally proximal to sepsis onset or incorporate clinical severity scores originally designed to identify established organ dysfunction, such as SOFA, qSOFA, or SIRS (1, 6, 7). Similar concerns have been raised in prior reviews, which highlight that inadequate temporal separation between predictors and outcomes may inflate performance estimates and obscure true predictive capability (8, 15).

High reported discriminatory performance, frequently expressed as AUROC values exceeding 0.9, was observed across multiple model categories (16–18, 27). However, AUROC alone provides limited insight into clinical utility, particularly in low-prevalence conditions such as sepsis. In these contexts, even models with high AUROC may yield a high number of false positive alerts, leading to increased alert burden and potential alert fatigue among clinicians (8, 10).

Clinically meaningful evaluation requires the inclusion of threshold-dependent metrics such as sensitivity, specificity, positive predictive value, and false alert rates, which were inconsistently reported across studies (8, 10). In addition, few studies incorporated approaches such as decision curve analysis to assess net clinical benefit across different threshold probabilities. The absence of these evaluations limits the ability to determine whether model implementation would improve clinical decision-making or patient outcomes in real-world settings (8).

Validation strategies further constrain generalizability. Most studies relied on internal validation, with relatively few conducting external or multicenter validation (11, 30, 31). Although recent work has begun to explore international validation using large datasets, substantial variability in performance across sites persists (31, 33). Temporal validation and evaluation of dataset shift were rarely addressed, despite their importance for sustained clinical deployment.

Despite increasing model sophistication, including deep learning and multimodal architectures (4, 18, 23, 28), evidence of clinical impact remains limited. Only a small number of studies evaluated prospective implementation or measured effects on patient outcomes or workflow integration (19, 20). Proprietary decision support systems showed greater implementation maturity in selected settings but often lacked transparency and independent evaluation (10, 38).

Recent studies have also begun to evaluate artificial intelligence models in more clinically relevant settings, including prospective validation and real-time deployment within intensive care workflows. However, these efforts remain limited, and most models have not progressed beyond retrospective evaluation, highlighting a persistent gap between methodological development and real-world implementation (10, 11, 19).

The geographic concentration of studies in high income settings further limits external validity and raises concerns regarding equity and bias (2, 3). Models trained in resource rich intensive care units may not generalize to settings with limited infrastructure or different clinical workflows, potentially exacerbating existing disparities if adopted without context specific validation (8, 34, 37).

Taken together, these findings indicate that future research should prioritize clear temporal outcome definitions, robust external and prospective validation, transparent reporting of clinically actionable metrics, and systematic evaluation of real world implementation. Addressing these dimensions is essential to move artificial intelligence based sepsis detection systems from proof of concept toward clinically meaningful and equitable deployment in intensive care practice.

### Limitations

This scoping review has several limitations that should be considered when interpreting the findings. First, consistent with scoping review methodology, no formal risk of bias assessment was conducted. Although key methodological features such as validation strategies, outcome definitions, and potential sources of bias were systematically examined, the absence of a structured quality appraisal may limit the ability to assess the overall strength of the evidence.

Second, the inclusion of studies was restricted to peer-reviewed publications indexed in major databases, and grey literature was not systematically searched. This approach may have introduced publication bias, as studies with negative or inconclusive results are less likely to be published.

Third, substantial heterogeneity was observed across included studies in terms of sepsis definitions, model architectures, data sources, prediction horizons, and validation approaches. This heterogeneity limited direct comparability between studies and precluded quantitative synthesis.

Fourth, most included studies were retrospective and conducted in single-centre or high-income settings, which may limit the generalisability of findings to other clinical contexts, particularly in low- and middle-income countries.

Finally, although efforts were made to ensure consistency in study selection and data extraction through independent review and consensus procedures, the possibility of subjective interpretation cannot be completely excluded, particularly in relation to the classification of analytical intent and the assessment of methodological features.

## Conclusions

Artificial intelligence based models for early sepsis detection in intensive care units demonstrate substantial analytical potential, with many studies reporting high discriminatory performance. However, this scoping review shows that apparent performance gains frequently coexist with important methodological limitations, including inconsistent outcome definitions, limited external and temporal validation, and insufficient assessment of clinical utility.

A central challenge across the current literature is the persistent ambiguity between prediction and detection. Many models labeled as early prediction systems incorporate predictors that reflect established physiological deterioration or organ dysfunction, thereby limiting their capacity to provide clinically actionable early warning within a meaningful intervention window. Consequently, high performance metrics alone are insufficient to justify clinical adoption, particularly in low prevalence conditions such as sepsis, where false alerts may have significant implications for patient safety and workflow efficiency.

The integrated synthesis further indicates that most models remain at early stages of technological readiness, with scarce prospective evaluation and limited evidence of real world implementation or impact on patient outcomes. In addition, the concentration of evidence in high income settings raises concerns regarding generalizability and equity, underscoring the need for context specific validation prior to widespread deployment.

Future research should shift emphasis from incremental performance optimization toward methodological rigor and clinical relevance. Priorities include clear temporal separation between predictors and outcomes, robust external and prospective validation, transparent reporting of clinically interpretable performance metrics, and systematic evaluation of workflow integration and clinical impact. Addressing these dimensions is essential to advance artificial intelligence based sepsis detection systems from proof of concept toward safe, effective, and equitable tools that meaningfully support decision making in diverse intensive care environments.

## Data Availability

The original contributions presented in the study are included in the article/Supplementary Material, further inquiries can be directed to the corresponding author.
